# Correlates of aggressive behavior in the moroccan context

**DOI:** 10.1192/j.eurpsy.2023.641

**Published:** 2023-07-19

**Authors:** A. Tounsi, Z. Bencharfa, F. Azraf, F. Laboudi, A. Ouanass

**Affiliations:** Arrazi university psychiatric hospital, Sale, Morocco

## Abstract

**Introduction:**

Violence among the mentally ill population has long been a subject of stigma, and controversy. Clinicians’ ability to assess the violent potential is still limited.

**Objectives:**

The objective of this work is to identify the positively correlated indicators of aggressive behavior in patients admitted to hospital emergency departments.

**Methods:**

It is a retrospective and descriptive paper based on the records of patients admitted to the emergency department of Arrazi University Psychiatric Hospital in Salé during a one-month period.

The psychiatric diagnosis was formulated using the DSM-5 diagnostic criteria and Violent behavior was assessed using the Modified Overt Aggression Scale (MOAS). The existence of aggressive behavior was defined by a MOAS score ≥3.

We used SPSS 15 to analyse results

**Results:**

Sixty-five case files were selected during the study month. The average age was 35.3 (19;64).

The mean of our sample MOAS aggression scale score was 31.5 [0; 79] and 90% of patients had a score ≥ 3 (image 1)

Among the 65 admissions, heteroaggressive risk was the most frequent reason for hospitalization (N=53), followed by psychomotor excitement. IMAGE 2

Statistical analysis revealed a significant association between high MOAS scores and substance use, history of suicide attempt, educational level and socioeconomic level

**Image:**

**Figure:**
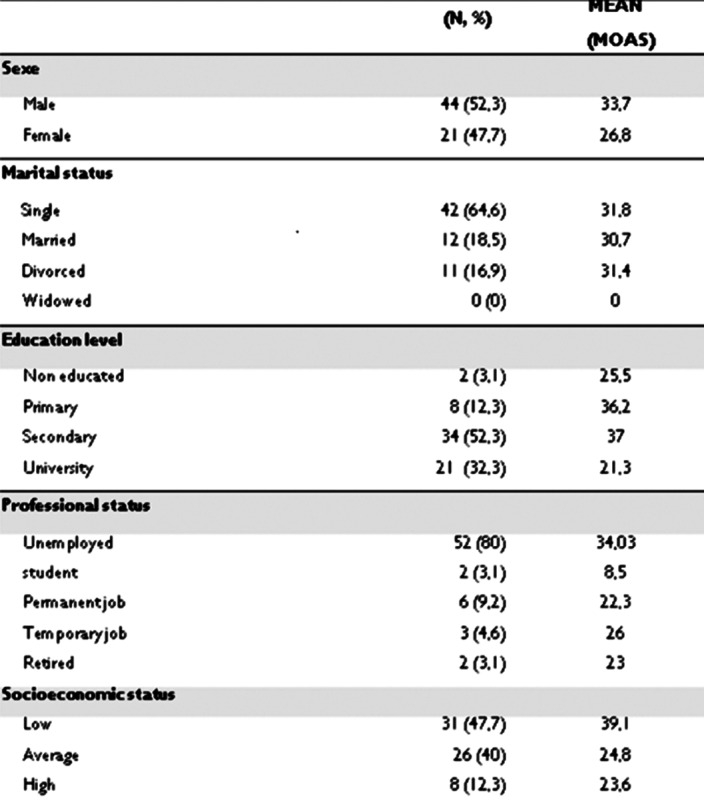

**Image 2:**

**Figure:**
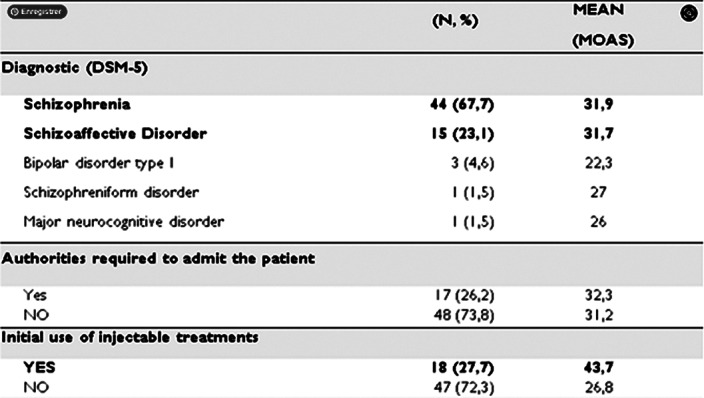

**Conclusions:**

In the current research, the prevalence of aggressive behavior was high among these patients, which may be due to the conditions of psychiatric hospitalization in our region, which is often reserved for the most serious and dangerous patients.

**Disclosure of Interest:**

None Declared

